# The Role of the Anterior Cingulate Cortex and Insular Cortex in Suicidal Memory and Intent

**DOI:** 10.7759/cureus.16335

**Published:** 2021-07-12

**Authors:** Sara Parisi, Ravindi Gunasekara, Cecilia Canale, Felicia Hasta, Heela Azizi

**Affiliations:** 1 Psychiatry, American University of Antigua College of Medicine, Coolidge, ATG; 2 Psychiatry, Medical University of the Americas, Charlestown, KNA

**Keywords:** suicide, cingulate cortex, insular activity, suicidal behavior, hypoxia

## Abstract

Research on the brain regions involved in suicidal behavior has led to the identification of a number of regions as being implicated in the neurobiology of suicide. Some of the brain regions identified have a clear correlation between the levels of activity and suicidal behavior. For some regions, such as the insular cortex and anterior cingulate cortex (ACC), both hyperactivity and hypoactivity have been correlated with suicidal behavior. More studies are needed to characterize more clearly the activity of the insular cortex and ACC when it comes to suicidal behavior. The case report presented adds to the literature seeking to clarify the correlation between ACC and insular activities and suicidal behavior. Structural damage of the insular region and ACC, thus leading to a decline in their activity level, was found in a patient who had resolution of suicidal intent after hypoxic brain injury. We discuss the significance of our findings for the neurobiology of suicide.

## Introduction

The neurobiology of suicide has been the focus of research over the past few decades. A significant portion of the research has been undertaken to identify the brain regions associated with suicidal behavior. In these studies, researchers tried to identify the direction of change in brain regions in patients demonstrating suicidal behavior, whether it be increased activity, decreased activity, increased volume, or decreased volume [1-5]. Such methods have identified changes in the structure and function of the orbitofrontal and dorsolateral areas of the prefrontal cortex (PFC) in suicidal patients. Alterations in activity in the anterior cingulate cortex (ACC), dorsolateral parts of the PFC and striatum in depressive patients with suicidal behaviors have been reported [1].

In recent years, neuroimaging methods, such as magnetic resonance imaging (MRI), have been utilized to study the directional change in the activity of the brain regions identified for suicidal behavior and their correlation. It was shown that there is a decreased activation in the ventral PFC during cognitive control tasks in patients with suicidal behavior [6]. Meanwhile, these patients may have either increased or decreased volume and activity in certain brain regions, such as the insula. In addition, studies on the ACC have shown traces of dorsal and ventral ACC activation alterations in suicidal behavior, but the direction of alterations (increased or decreased) seems to be complex and dependent on task condition and stimulus type [6]. More studies would therefore be needed to clarify the direction of changes in these brain regions of patients demonstrating suicidal behavior.

This case involves a patient with a history of multiple suicide attempts where the patient demonstrated a resolution of suicidal ideation and intent following hypoxic brain injury involving the medial anterior frontal cortex, insular cortex, ACC, cerebellum and medial temporal regions. The significance of these findings in understanding the neurobiology of suicide is discussed, and it is for this reason that we present this case report with the goal of advancing our understanding of the direction of alteration of brain regions in a patient with suicidal behavior.

## Case presentation

The case presented here is of a 47-year-old man with a psychiatric history of major depressive disorder (MDD), three prior suicide attempts, anxiety disorder and with no medical comorbidities or substance use disorders. The patient showed a progressive exacerbation of his MDD over a period of one year after losing his job in construction, and increasing marital distress. He was found hanging from a rope with a suicide note, by family members. Family accounts indicated his last interaction was three minutes prior to being found hanging. Emergency Medical Services (EMS) was activated after the patient was successfully released from the hanging rope but was found to be unresponsive. EMS indicated that on arrival, they found the patient in a decorticate position with pupils fixed, and in state of a cardiac arrest. He was successfully resuscitated but remained unconscious.

On arrival in the emergency room, routine examination and radiological workup showed no evidence of carotid intimal dissection, thrombus formation, tracheal stenosis, cervical spinal injury or spinal cord injury. However, there was superficial soft tissue injury on the neck along the tracks of the rope used for hanging. He was admitted to the intensive care unit (ICU), with interventions including mechanical ventilation with a target oxygen saturation of 94%-98% and a target arterial blood gas pO_2_ of 70-100 mmHg and pCO_2_ of 40 mmHg and monitoring for complications such as acute respiratory distress syndrome (ARDS) and further cardiac arrest and raised intracranial pressure. His course in the ICU indicated he was maintained at a target systolic blood pressure (SBP) of 100 mmHg with an intermittent need for a dose of adrenaline not exceeding 20 mcg/min. His ICU course was complicated by the development of aspiration pneumonia for which he was managed with 2 g of cefepime intravenously every 12 hours for seven days and vancomycin for a total of 2 g divided as 500 mg every six hours. He was eventually successfully weaned off mechanical ventilation. A repeat examination for complications of his suicide attempt indicated no evidence of neurologic sequelae such as hemiplegia, central cord syndrome, or spinal cord injury. Of note, during his mental status examination, he was oriented to person and time but not situation, as he was unable to recall the events of his admission to the hospital. He remained depressed over work and marital problems. When attempts were made to orient him to the likely suicide attempt including the suicide note, he was reported to have expressed that it was not possible that he could have resorted to hanging himself. He reported that if he had harmed himself, he would be remorseful of the event and seek methods to prevent it; however, he did not recall attempting to harm himself. He expressed that although he was aware of his stressors and depressed mood, he was unable to think through a process that would lead him to draw a conclusion to end his own life.

He was admitted to inpatient psychiatry service for the ongoing depressed mood, poor recollection of his suicide attempt. On initial psychiatric evaluation in inpatient psychiatry, he continued to report depressed mood, anhedonia, poor sleep and poor appetite changes. He could not recall taking any action as a result of his depressed mood and reported he coped with his depression by keeping himself busy with work. The patient continued to express the egodystonic nature of suicidal thoughts or actions. He reported he had no desire or impulse to harm himself and could not have hanged himself prior to coming to the hospital. In addition, he reported no recollection of his prior suicide attempts and appeared distressed when he was told he may have tried to harm himself in the past. When he was shown the scars around his neck, he was unable to recall how they had happened. He expressed that his egosyntonic response to his stressors was to utilize his family support and to use religious means to cope with his stressors. Collateral information obtained from family indicated that this would be his fourth suicide attempt. Previous attempts involved overdose on medications and he was managed on the medical floors with observation only. He also had several hospitalizations for depression and suicidal ideations that he openly expressed to his family. According to family members, after the prior instances of suicide attempt, he was able to acknowledge his attempt and express remorse for his attempts, unlike his current attempt. In addition, he sought treatment for his mental illness willingly following those attempts, and was managed on Lamictal 50 mg twice daily. The family reported they had been supportive of him following the attempts and he often opened up about his feelings without reservations. There was no reported high emotionality or frustration by family members expressed towards his mental illness or suicide attempts and they were surprised he was not able to recollect these attempts. According to them, this was the first time he could not remember his suicide attempts, as he openly discussed them in the past and sought support from family about them.

On mental status examination upon hospitalization, the patient was alert but not oriented to time, place, or person. The patient was disoriented, unkempt, and exhibited psychomotor retardation on examination. During his stay, he alternated between recognizing being in a hospital and believing he was at a construction site where he had to perform different working tasks. No focal neurologic deficits were noticed on the neurologic exam except for marked bilateral intention tremor. In light of his difficulty with recollection, his mental status was assessed for which he scored 15/30 on the Montreal Cognitive Assessment (MoCA) exam with significant difficulties especially in word recollection and scored 4/30 on the Mini-Mental State Exam (MMSE). The Hamilton Depression Rating Scale (HAM-D) score was 26.

**Figure 1:**
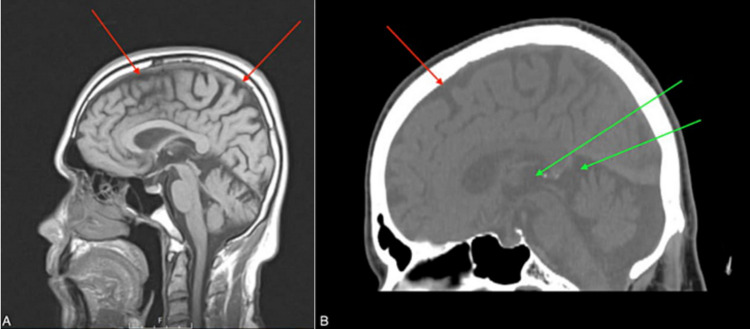
Sagittal T1 diffusion-weighted head MRI. (A) Hypoattenuation in the cortex suggests sulci enlargement and generalized cortical atrophy (red arrows). (B) Further evidence of cortical volume loss (red arrow) and concomitant ventricular enlargement (green arrows)

**Figure 2:**
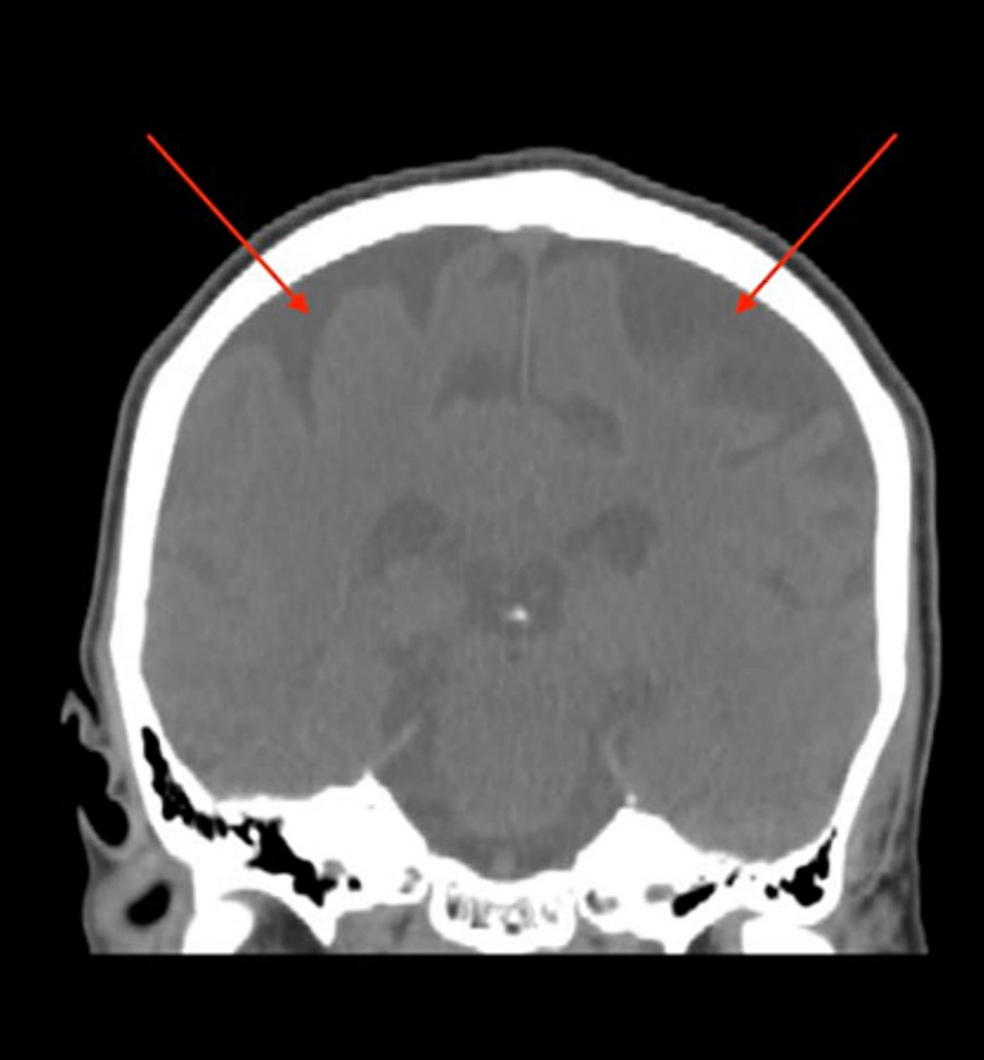
Coronal T1 diffusion-weighted head MRI, with hypoattenuation representing marked cortical atrophy (red arrows)

Routine workup during admission including levels of vitamin B12, folate, vitamin B1, thyroid stimulating hormone (TSH), and rapid plasma reagin (RPR) was within normal limits. Urine toxicology was also negative. Non-contrast head computed tomography (CT) and MRI were also performed. There was no evidence of acute bleeding, mass, or midline shift intracranially. The results of the imaging studies indicated that the ventricular system and subarachnoid spaces were prominent, suggesting cerebral atrophy. More specifically, beginning in the frontal cortex, there was ischemic injury with volume loss in the medial frontal cortex region. The lateral and medial ventral prefrontal cortex were preserved with no signs of structural injury due to ischemia of the region. The dorsal PFC was also preserved. Extensive volume loss was noted in the medial temporal lobe structures including left hippocampus, lateral temporal lobes, anterior frontal poles, the parietal lobes, insular cortex and the cerebellum. These findings are shown in Figures 1, 2.

His treatment plan involved the management of his depressed mood, as well as exploring and improving his insight into his suicidal behavior and developing alternative coping strategies during stressful life events. During the course of hospitalization on the psychiatric floor, he was commenced on sertraline 50 mg that was titrated to 200 mg due to subtherapeutic doses. He saw slight improvement in his mood, but still remained depressed and his medication was augmented with aripiprazole starting at 5 mg PO daily and increased to 10 mg over a period of one week, again due to subtherapeutic doses. No side effects to either medication were reported. At the end of the third week, he showed a response to treatment with an HAM-D score of 18, although he still had neurovegetative features of poor sleep. He was prescribed trazodone 150 mg PO hs to augment antidepressant effect and for insomnia. In addition, he was able to discuss his stressors and his reasons for depression much more clearly. However, he continued to maintain no recollection of his suicide attempt that led to his current admission, or any prior suicide attempts. He was still able to recollect other details of his life unrelated to suicide. He acknowledged the distressing nature of his stressors, but insisted he utilizes spirituality as coping strategies, and will continue to utilize the same for coping. He externalized any act of suicide by any person as being the work of outside forces based on his religious beliefs. The patient expressed that such an act would not follow from his thought processes. When shown the scar around his neck, he stated it may have been a work-related injury or some skin disorder. He also insisted no recollection of prior suicidal thoughts or actions. He was discharged home, with improvement in his depressed mood, to his family who provided support and additional nursing services. He denied any suicidal ideations, plans or intent, and continued to report no recollection of his recent or prior suicide attempts.

## Discussion

The case presented is of a patient with a recurrence of a major depressive episode accompanied by a suicide attempt. It was his fourth suicide attempt and the most severe compared to previous attempts. He had a significant hypoxic damage to the brain involving the temporal cortex, frontal cortex, and subcortical structures secondary to the suicide attempt. The patient presented had no recollection of his suicide attempt and displayed no further suicidal ideation immediately after medical stabilization. The patient suffered from memory loss of the suicidal attempt and all memories related to prior suicide attempts. Antidepressants are known to resolve suicidal ideations, but the patient was not given antidepressants prior to the resolution of suicide ideation [7]. Sertraline, trazadone, and aripiprazole were started only after the resolution of suicidal ideations and the loss of memory of suicide attempt were noted. In addition, he continued to have a depressed mood, even though the accompanying suicidal ideations had resolved. The resolution of the stressors that prompted his suicide attempt could have also led to a resolution of suicidal ideation, but patient’s stressors remained active plus he had a recollection of his stressors. Thus, we believe there may be an association between the hypoxic brain injury in this patient, and the loss of his suicidal memory and intent. The brain regions impacted by hypoxic injury in this patient include the medial frontal cortices, cerebellum, the hippocampus, the ACC, the insular and the parietal lobe.

There have been studies showing increased and decreased activity correlating with suicidal behavior and the involvement of the anterior cingulate gyrus. In terms of the patient's inability to recall his suicide attempt, Reisch et al. used functional MRI (fMRI) during the presentation of autobiographical scripts extracted from personal narratives reactivating patients' memories of a recent episode of attempted suicide [3]. Brain activation was measured during three recalled conditions: mental pain, suicide action and neutral activity. They found recall of suicide action was associated with higher activity in the medial PFC, the ACC, and the hippocampus. In addition, the direction of change in this patient was volume loss in the anterior cingulate gyrus region. A review of prior studies had been unclear in the association between the anterior cingulate gyrus changes and suicidal behavior. In one of the studies that seemed to be consistent with our findings, Sublette et al. compared regional cerebral metabolic rates of glucose (rCMRglu) determined by [^18^F]-fluoro-2-deoxyglucose positron emission tomography (FDG-PET) in suicide attempters and non-attempters [2]. They looked at brain regions in which relative rCMRglu differed between depressed participants who attempted suicide within two years before or after scanning (attempters), compared with those who never attempted suicide (non-attempters). The regions in which attempters showed changes greater than non-attempters after placebo included the anterior cingulate, caudate, putamen, inferior frontal, insula and medial frontal. Similar findings were also reported by Jollan et al. who studied the blood oxygenation level-dependent fMRI activity in right-handed men with a past history of MDD, comparing suicide attempters with non-attempters [4]. In addition, Oquendo et al. also found increased rCMRglu in cerebellum bilaterally in right-handed high-lethality compared with low-lethality attempters [5]. It has been suggested that the cerebellum, which has reciprocal connections with various limbic structures, may play a role in the regulation of emotions such as happiness, anger, and sadness. In this same study, suicide attempters showed greater activity in the right lateral orbitofrontal cortex in response to prototypical angry versus neutral faces, and greater activity in the right anterior cingulate gyrus in response to mild happy versus neutral faces. An additional finding in our patient was hypoxic injury to the cerebellum with cerebellar signs seen on neurological examination. This is consistent with findings on increased cerebellar activity in suicidal patients reported by Oquendo et al. [5].

Throughout the literature, the change in volume and/or activity in certain brain areas has been linked to patients demonstrating suicidal behavior, but what has been missing so far is a clear and unequivocal correlation between the two [6].

This case reports some interesting links between suicidal behavior and hypoxic injury to different brain regions (Table 1). According to our literature review, 57% of the articles talked about the PFC, 71% about the ACC, and 28% about the insula; 28% reported an increase in the cerebellum activity and only 14% discussed the striatum, hippocampus, caudate, putamen, inferior frontal cortex and medial frontal cortex activity. The patient in our case, with a history of multiple suicide attempts, demonstrated a resolution of his suicidal ideations and intent following hypoxic brain injury involving the medial anterior frontal cortex, insular cortex, ACC, cerebellum and medial temporal regions.

**Table 1:** Increase or decrease in activity in specific brain areas PFC: prefrontal cortex; ACC: anterior cingulate cortex; MFC: middle frontal cortex; IFC: inferior frontal cortex; N/A: not applicable

	Desmyter et al., 2011 [1]	Schmaal et al., 2020 [6]	Reisch et al., 2010 [3]	Sublette et al., 2013 [2]	Jollant et al., 2008 [4]	Oquendo et al., 2003 [5]
Dorsolateral PFC	↓	N/A	N/A	N/A	N/A	N/A
Orbitofrontal PFC	↓	N/A	N/A	N/A	↑	N/A
ACC	↓	↑/↓	↑	↓	↑	N/A
Striatum	↓	N/A	N/A	N/A	N/A	N/A
Ventral PFC	↓	↓	N/A	N/A	N/A	N/A
Insula	↓	↑/↓	N/A	↓	N/A	N/A
Median PFC	↓	N/A	↑	N/A	N/A	N/A
Hippocampus	N/A	N/A	↑	N/A	N/A	N/A
Caudate/putamen	↓	N/A	N/A	↓	N/A	N/A
IFC/MFC	N/A	N/A	N/A	↓	N/A	N/A
Cerebellum	↓	N/A	N/A	N/A	↑	↑

## Conclusions

With this study, we theorize that there may be an association between the structural damage of selected brain regions and the resolution of suicidal intent after hypoxic brain injury; however, further studies are needed to prove such a relationship. The brain regions impacted by hypoxic injury in our patient include the medial frontal cortices, the cerebellum, the hippocampus, the anterior cingulate cortex and the insular and the parietal lobe, all of which have been linked to a decrease in suicidal ideation in past studies. Other possible reasons that could have led to the resolution of the suicidal ideation and attempts were taken into consideration; antidepressants were not given prior to the resolution of suicidality, stressors remained active afterwards and he had a recollection of his stressors. Structural damage and hypofunctioning following a hypoxic injury to the ACC and the insula may lead to the loss of suicidal memory with a subsequent reduction in suicidal intent. The patient in this case was discharged home under family and additional nursing services, and reported improvement of depressed mood with continuous denial of any suicidal attempt or any recollection of his recent or past suicide attempts. Due to loss of follow-up, subsequent information could not be gathered from the patient, thus creating a limitation for the study. Further studies are needed to explore the significance of these findings for possible therapeutic management of patients with suicidal behavior.
